# Glial cells are affected more than interneurons by the loss of *Engrailed 2* gene in the mouse cerebellum

**DOI:** 10.1111/joa.13982

**Published:** 2023-11-27

**Authors:** Giulia Lazzarini, Alessandra Gatta, Vincenzo Miragliotta, Francesca Vaglini, Cristina Viaggi, Andrea Pirone

**Affiliations:** ^1^ Department of Veterinary Sciences University of Pisa Pisa Italy; ^2^ Department of Translational Research and of New Surgical and Medical Technologies University of Pisa Pisa Italy

**Keywords:** autism, cerebellum, engrailed, glia, interneurons, mice

## Abstract

Glial cells play a pivotal role in the inflammatory processes, which are common features of several neurodevelopmental and neurodegenerative disorders. Their major role in modulating neuroinflammation underscores their significance in these conditions. Engrailed‐2 knockout mice (*En2* ^*−/−*^) are considered a valuable model for autism spectrum disorder (ASD) due to their distinctive neuroanatomical and behavioral traits. Given the higher prevalence of ASD in males, our objective was to investigate glial and interneuron alterations in the cerebellum of *En2* ^*−/−*^ mice compared with wild‐type (WT) mice in both sexes. We employed immunohistochemical analysis to assess cell density for all cell types studied and analyzed the area (A) and shape factor (SF) of microglia cell bodies. Our findings revealed the following: (a) In WT mice, the density of microglia and astrocytes was higher in females than in males, while interneuron density was lower in females. Notably, in *En2*‐mutant mice, these differences between males and females were not present. (b) In both male and female *En2* ^*−/−*^ mice, astrocyte density exceeded that in WT mice, with microglia density being greater only in females. (c) In WT females, microglia cell bodies exhibited a larger area and a lower shape factor compared to WT males. Remarkably, the *En2* mutation did not appear to influence these sex‐related differences. (d) In both male and female *En2* ^*−/−*^ mice, we observed a consistent pattern: microglia cell bodies displayed a larger area and a smaller shape factor. Given the ongoing debate surrounding the roles of glia and sex‐related factors in ASD, our observations provide valuable insights into understanding how an ASD‐associated gene *En2* affects specific cell types in the cerebellum.

## Graphical abstract

Engrailed‐2 knockout mice (En2^−/−^) are a model for autism spectrum disorder (ASD), moreover the role of the cerebellum in ASD has been established. In this context, we conducted an immunohistochemical analysis to investigate whether changes in glial and/or interneurons occur in the cerebellum of En2^−/−^ mice compared to wild‐type in both males and females.

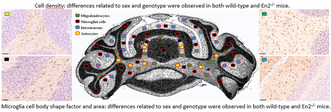

## 1 INTRODUCTION

Neuroinflammation is a condition common to several neurodevelopmental and neurodegenerative disorders, including Alzheimer's, Parkinson's, amyotrophic lateral sclerosis, and autism. However, whether it is an effect, or a triggering cause remains far from completely understood (Cheataini et al., 2023; Zhang et al., 2023).

In recent decades, a wealth of data has demonstrated the involvement of glial cells in neuroinflammation and, consequently, in central nervous system (CNS) disorders (Patani et al., 2023; Woodburn et al., 2021). Under normal conditions, glia is crucial for neuronal survival and function, with their physiological roles encompassing phagocytosis, synaptic pruning and modulation, programmed cell death, neuronal plasticity, trophic support, neurotransmitter uptake, and myelination. Aberrations in these functions may lead to cognitive dysfunction in neurological diseases (Fyfe, 2022; Patani et al., 2023; Rahman et al., 2022; Salter & Stevens, 2017).

In particular, the activation of astrocytes and microglia appears to be linked to autism spectrum disorders (ASD), abnormal microglial counts and morphology have been described in the brains of individuals with autism as well as in brains from ASD animal models (Petrelli et al., 2016; Prata et al., 2017; Vargas et al., 2005).

While little information related to glial cells in the cerebellum has been reported to date, it is known that mice lacking the *En2* gene (*En2* ^*−/−*^ mice) are considered a valuable neurobiological model due to the display of neuroanatomical and behavioral traits relevant to ASD (Benayed et al., 2009; Cheh et al., 2006; Gharani et al., 2004).

*En2* is one of the genes that regulate cerebellar development, and *En2* ^*−/−*^ mice exhibit an altered cerebellar foliation pattern (Millen et al., 1994), cerebellar hypoplasia (Joyner et al., 1991), and a reduction in Purkinje cell numbers (Kuemerle et al., 1997). Traditionally, the cerebellum has been described as a structure primarily involved in motor learning and coordination. However, it has recently been recognized for its role in cognitive processing and social behavior, thus establishing its involvement in ASD (Brielmaier et al., 2012; Hoche et al., 2016; Mapelli et al., 2022).

In this context, we conducted an immunohistochemical analysis to investigate whether changes in glial and/or interneurons occur in the cerebellum of 6‐week‐old *En2* ^*−/−*^ mice compared with wild‐type (WT) mice in both males and females.

## 2 MATERIALS AND METHODS

### 2.1 Mice

No animals were killed for the purpose of this study. Paraffin‐embedded cerebellum here used was from previous research performed on the telencephalic reticular nucleus of mice lacking *En2* (*En2* ^*−/−*^ mice; Pirone et al., 2020).

The original *En2* mutants (mixed 129Sv × C57BL/6J and outbred genetic background) were crossed at least five times into a C57BL/6J background. Heterozygous mating (*En2* ^*+/−*^ *× En2* ^*+/−*^) was used to generate the *En2* ^*+/+*^ (WT) and *En2* ^*−/−*^ (KO‐mice) littermates used in this study. Assessment of the *En2* ^*−/−*^ status in adult mice was confirmed by the absence of *En2* ^*−/−*^ by tail DNA PCR genotyping according to the protocol available on the Jackson Laboratory website (www.jax.org; mouse strain En2tm1Alj). KAPA Mouse genotyping Kits (KAPA biosystems), consisting of KAPA Express Extract and KAPA2G Fast Genotyping Mix was used for the routine extraction and amplification of DNA for mouse genotyping. Experiments were conducted in conformity with the European Directive of 24 November 1986 (86/609/EEC and 2010/63/UE) and in agreement with the Italian DM26/14. Experiments were approved by the Ethics Committee of the University of Pisa.

Twelve females (6 WT, 6 *En2* ^*−/−*^) and 11 males (6 WT, 5 *En2* ^*−/−*^; weight = 18–30 g) 6 weeks old were included in the study as shown in Table 1.

**TABLE 1:** Animal groups used in this study.

Group	Age (weeks)	Sex	WT	*En2* ^*−/−*^	Total no. animals/group
1	6	M	6	5	11
2	6	F	6	6	12

### 2.2 Immunohistochemistry

Immunohistochemistry was performed on serial paraffin sections (5 μm) using a mouse monoclonal anti‐Parvalbumin (PV) as interneurons marker, a rabbit polyclonal anti‐Glial fibrillary acidic protein (GFAP) as an astrocytes marker, rabbit polyclonal anti‐oligodendrocyte transcription factor 2 (Olig‐2) as a oligodendrocytes marker, and rabbit polyclonal anti‐ionized calcium‐binding adapter molecule1 (Iba1) as a microglial marker, antibodies details are reported in Table 2. Epitope retrieval was carried out at 120°C in a pressure cooker for 5 min with a Tris/EDTA buffer, pH 9.0. Sections were pretreated with 1% H_2_O_2_ in PBS for 10 min to quench endogenous peroxidase activity, then rinsed with 0.05% Triton‐X (TX)‐100 in PBS (3 × 10 min) and blocked for 1 h with 5% normal horse serum (PK‐7200, Vector Labs, Burlingame, CA) in PBS. Serial sections were incubated overnight at 4°C in a solution containing the primary antibodies with 2% normal horse serum and 0.05% TX‐100 in PBS. Sections were then rinsed in PBS, (3 × 10 min), followed by incubation with biotinylated anti‐mouse IgG (for anti‐PV) or biotinylated anti‐rabbit IgG (for anti‐GFAP, anti‐Olig‐2, and anti‐Iba1) then with ABC reagent (Vectastain Kit, PK‐7200, Vector Labs, Burlingame, CA). Sections were again rinsed in PBS, for 3 × 10 min. Staining was visualized by incubating the sections in diaminobenzidine (sk‐4105, Vector Labs) solution. The specificity of immunohistochemical staining was tested by replacing either the primary antibodies, anti‐mouse IgG, or the ABC complex with PBS or nonimmune serum. Under these conditions, staining was abolished. Furthermore, the specificity of the antibodies had already been tested in previous studies (Antibody Registry https://antibodyregistry.org/).

**TABLE 2:** Antibodies.

Antibody	Immunogen	Manufacturing details	Antibody registry ID	Dilution
Anti‐PV	Purified frog muscle parvalbumin	Sigma, mouse monoclonal, P3088, Clone PARV‐19	AB 477329	1:2000
Anti‐GFAP	Glial fibrillary acidic protein	Sigma, rabbit polyclonal HPA056030	AB 2683015	1:2000
Anti‐Olig‐2	Recombinant mouse Olig‐2	Millipore, rabbit polyclonal, AB9610	AB 570666	1:500
Anti‐Iba1	Synthetic peptide (Iba1 C‐terminal sequence)	FUJIFILM Wako, rabbit polyclonal, 019–19,741	AB 839504	1:500
Biotinylated	Mouse IgG (H + L)	Vector Labs, Burlingame, horse, Cat. no. BA‐2000	AB 2313581	5 μg/mL
Biotinylated	Rabbit IgG (H + L)	Vector Labs, Burlingame, horse, Cat. no. BA‐1100	AB 2336201	5 μg/mL

### 2.3 Image acquisition and processing

Morphometry was performed on four coronal cerebellum sections 100 μm spaced per animal per marker. Only cells with a well‐recognizable body cell were counted.

Whole slide images were acquired with Nano Zoomer Hamamatsu slide scanner at a magnification of 20× with automatic focusing. The cell density (cells/mm^2^) of the immunostained cells was calculated using the software Stereo Investigator—Whole Slide Edition (MBF, Bioscience) considering our sections as a 2D system. To estimate the cell density, we employed the counting frame as the probe typically used to sample populations that are too large to be counted exhaustively. The ROI was traced using the contour tool. We set the size of the counting frame so that it contained a reliably countable number of cells (i.e., avoiding double counts or missing cells), and then the software allocated the probes in a systematic random sampling grid overlayed to the ROI (see Table 3 for details). Each counting frame included two exclusion lines (red) and two inclusion lines (green), a cell was counted if it was completely within the counting frame or if the cell body touched the inclusion lines.

**TABLE 3:** Settings parameters for each marker to calculate cell density.

Staining	ROI	Counting frame size (μm)
Anti‐Iba1	Whole section	150 × 150
Anti‐PV	Molecular layer	100 × 100
Anti‐GFAP	White matter	150 × 150
Anti‐Olig‐2	White matter	100 × 100

In addition to the cell density, for Iba1 immunoreactive cells the cell body area (A, μm^2^) and the shape factor (SF) were also calculated. SF is a measure of the roughness (SF = 1 indicates a round shape) of the selected object and is calculated by the following formula: 4π*Area/(convex hull perimeter)^2^. For each section 10 images at 40× magnification were randomly taken. Then, by semi‐automatic processing, the immunostained cell bodies were segmented and their area and shape factor were collected by using the Nis‐Elements Br Software (Nikon Instruments SPA, Calenzano, Italy).

**FIGURE 1:**
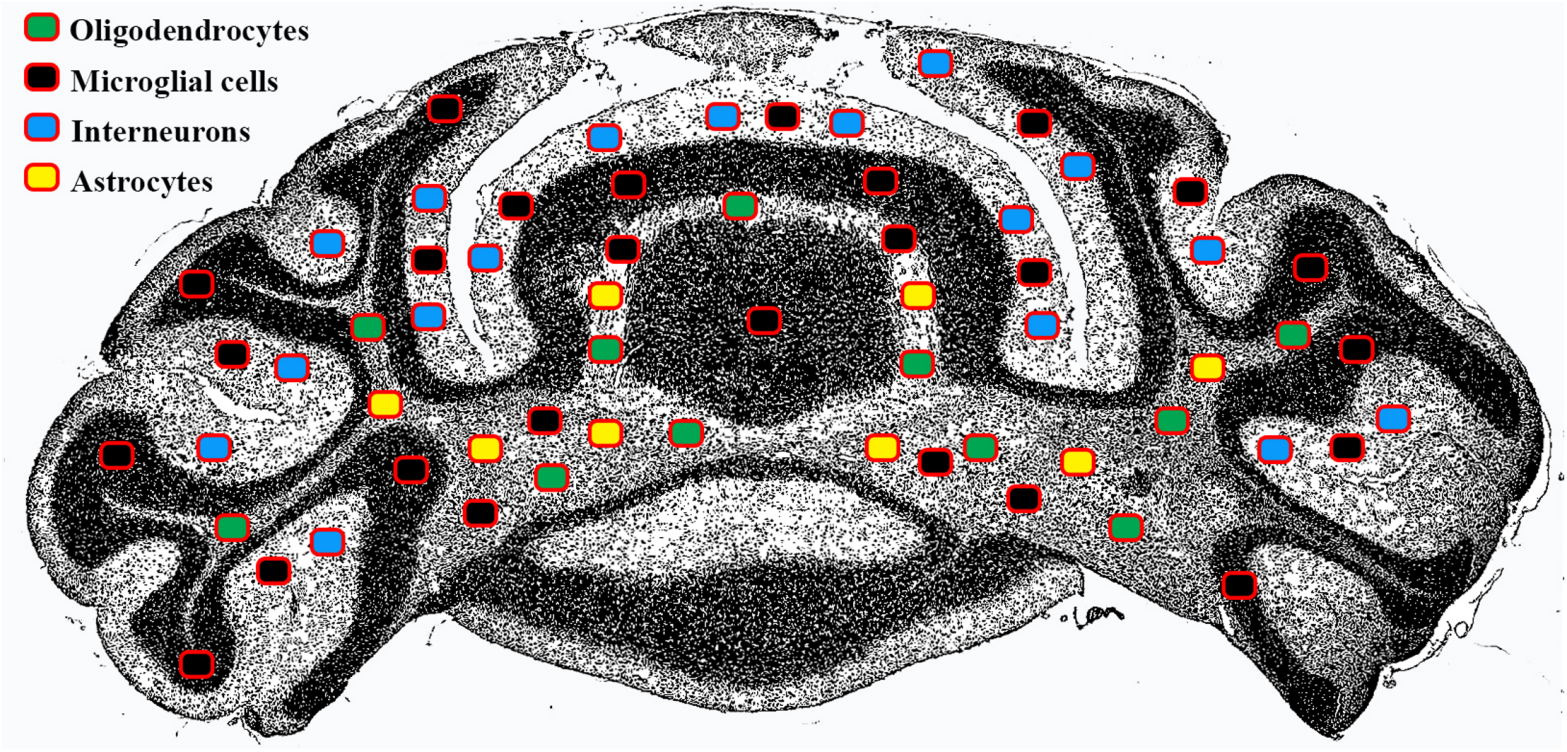
Drawing representing a cerebellum coronal section. The density (cells/mm^2^) of interneurons (blue) was calculated in the molecular layer. The density of astrocytes (yellow) and oligodendrocytes (green) was calculated in the white matter. The density of microglia (black) was calculated throughout the cerebellum.

The distribution of glial cells and interneurons was thus described throughout the cerebellum in both mutant and wild‐type mice (Figure 1). We calculated the density of interneurons (basket and stellate cells, marked with anti‐PV) in the molecular layer, the density of astrocytes (marked with anti‐GFAP) and oligodendrocytes (marked with anti‐Olig2) in the white matter, and the density of microglia (marked with anti‐Iba1) throughout the cerebellum.

### 2.4 Statistics

Data were tested for normality by using the Kolmogorov–Smirnov test. Due to the presence of not normally distributed values for some parameters, statistical analyses were carried out using the Mann–Whitney test, setting the significance at P < 0.05.

## 3 RESULTS

**FIGURE 2:**
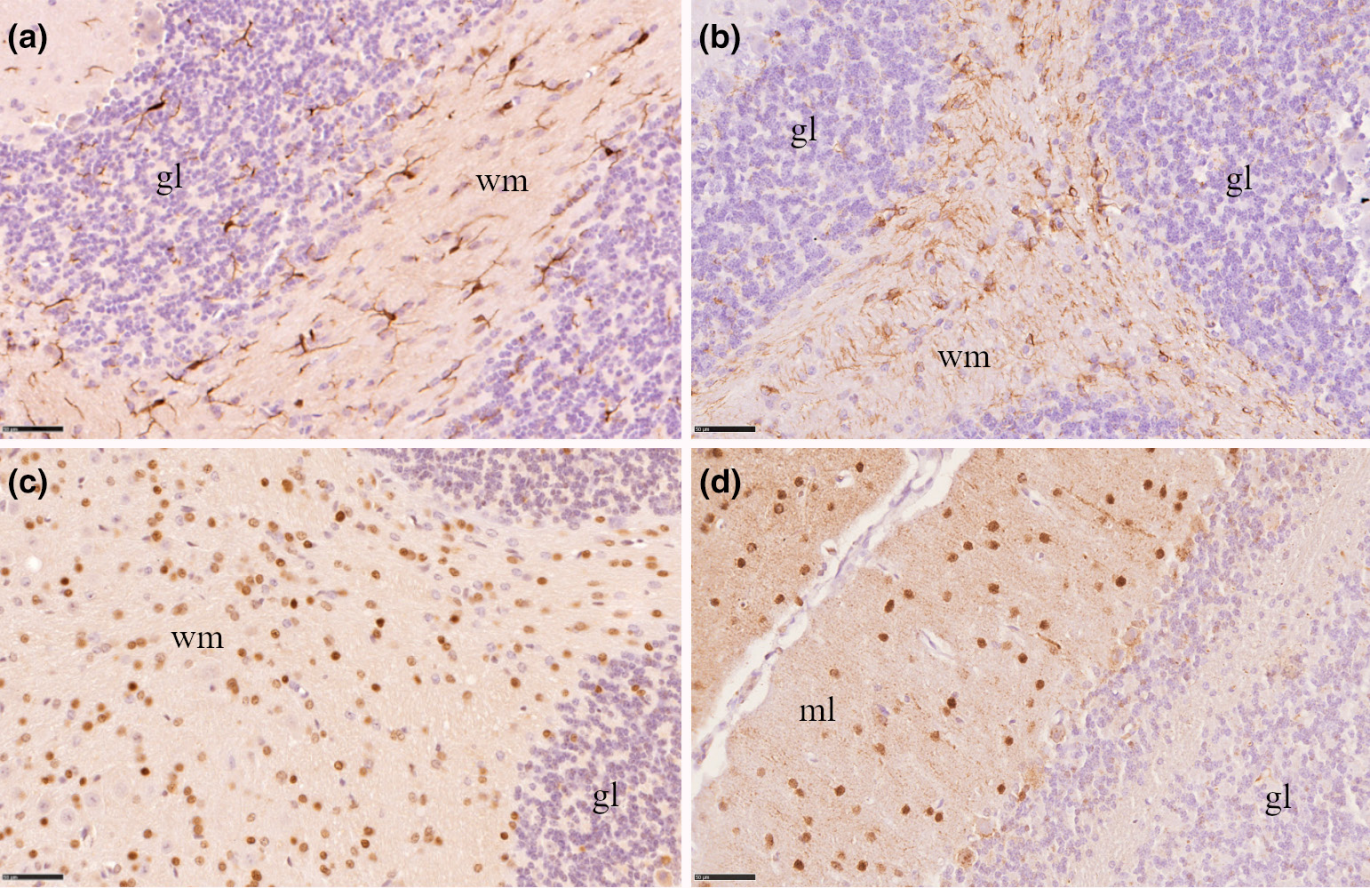
Immunohistochemical analysis showing the microglia (a), astrocytes (b), oligodendrocytes (c), and interneurons (d) distribution in the mice cerebellum. gl, granular layer; ml, molecular layer; WM, white matter. Scale bars = 50 μm.

Glial cells were distributed throughout the cerebellum in both wild‐type and mutant mice. Microglia and astrocytes showed their typical dendritic appearance; Iba1 immunoreactivity was found in both cell body and processes (Figure 2a); GFAP immunoreactivity was mostly confined to the cellular processes (Figure 2b); oligodendrocytes (Figure 2c) showed a round shaped immunoreactivity as well as interneurons (Figure 2d).

### 3.1 Cell density

**FIGURE 3:**
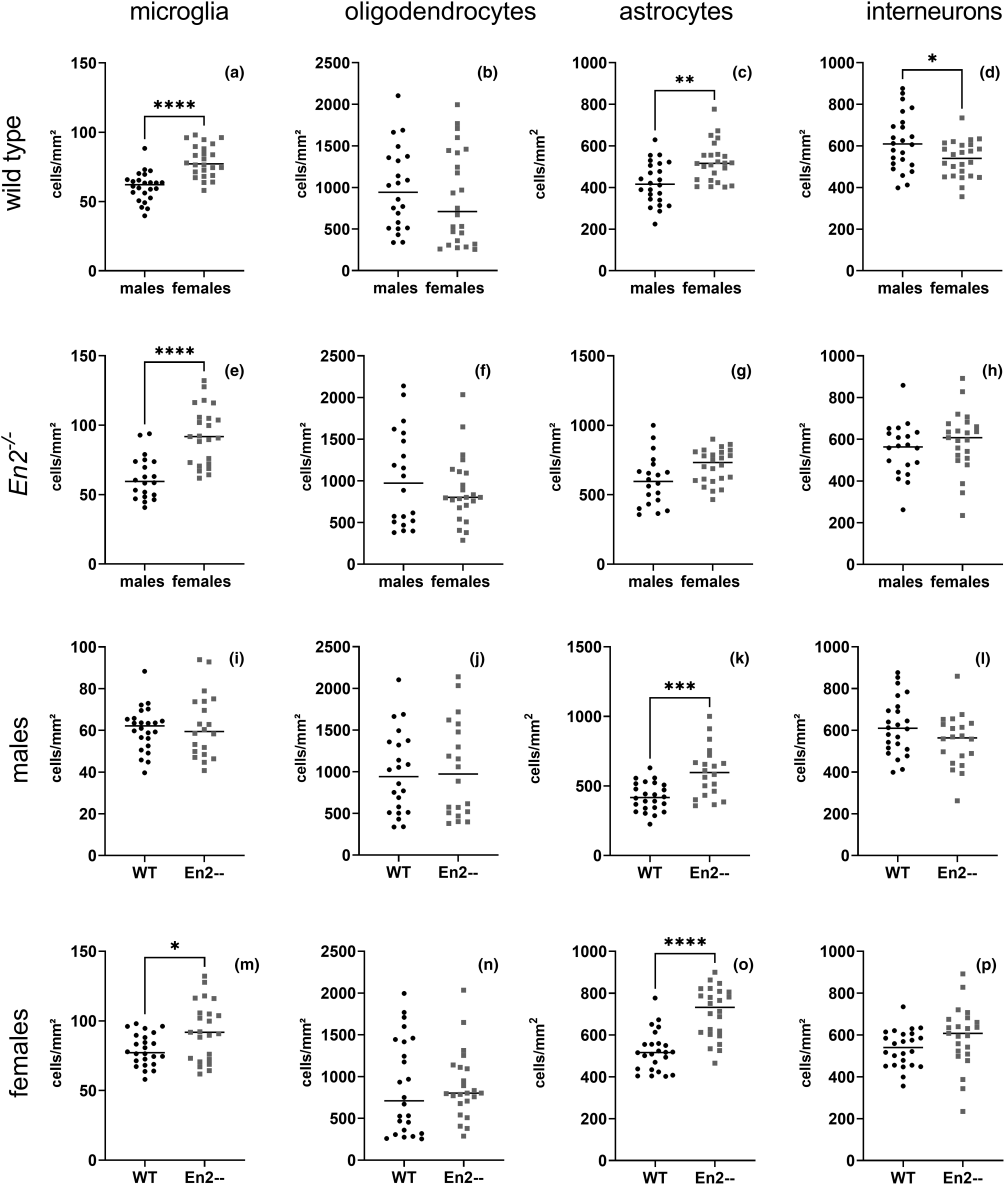
Scatter dot plots showing cell density differences of microglia, oligodendrocytes, astrocytes, and interneurons in males vs females (*En2* ^*−/−*^ and WT) and in WT vs *En2* ^*−/−*^ (males and females) mice. Asterisks indicate statistical significance: *p = 0.0298, **p = 0.0024, ***p = 0.0006, ****p < 0.0001. Statistical analyses were carried out using the Mann–Whitney test.

Figure 3a–d displayed sex‐related differences in WT mice, while differences were observed for microglia (Figure 3a), astrocytes (Figure 3c), and interneurons (Figure 3d) the same was not seen for oligodendrocytes (Figure 3b). Specifically, microglia and astrocytes showed a higher density (*p* < 0.0001 and *p* = 0.0024 respectively) in females while interneuron density was higher (*p* = 0.0355) in males. Analogously, Figure 3e–h showed sex‐linked differences in *En* ^*2−/−*^ mice where only microglia cell density was greater in females (*p* < 0.0001; Figure 3e). Oligodendrocyte, astrocyte, and interneuron cell density (Figure 3f–h) did not differ between males and females in mutant mice.

Figure 3i–l displayed differences related to the genotype in males: *En* ^*2−/−*^ males showed a statistically higher (*p* = 0.0006) astrocyte density compared to WT (Figure 3k); no differences were observed for microglia (Figure 3i), oligodendrocytes (Figure 3j) and interneurons (Figure 3l). In parallel, Figure 3m–p showed genotype‐related differences in female mice. Specifically, cell density was significantly higher in *En* ^*2−/−*^ females, compared with WT, for microglia (*p* = 0.0298; Figure 3m) and astrocytes (*p* < 0.0001; Figure 3o) while interneurons and oligodendrocytes did not display significant differences.

### 3.2 Microglia cell body features: Area and shape factor

**FIGURE 4:**
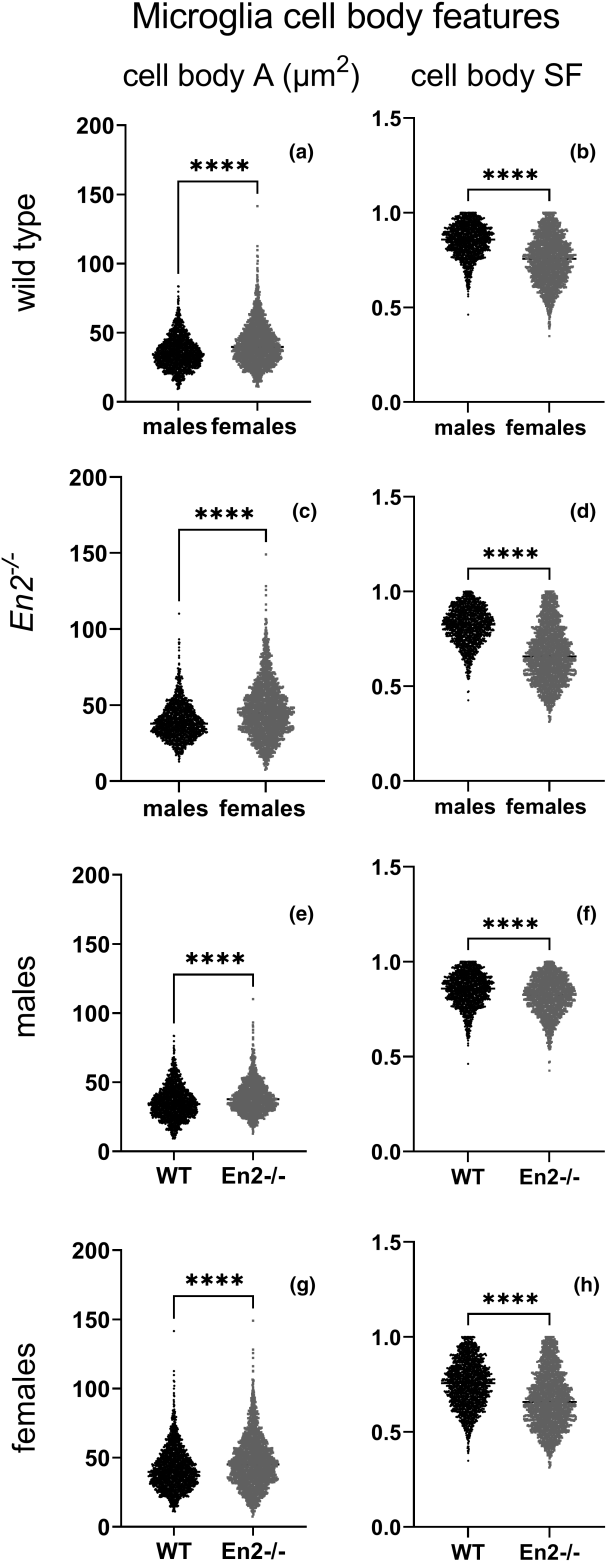
Scatter dot plots showing cell body area (A) and shape factor (SF) differences in males versus females (WT and *En2* ^*−/−*^) and in WT versus *En2* ^*−/−*^ (males and females) and mice. Asterisks indicate statistical significance: ****p < 0.0001. Statistical analyses were carried out using the Mann–Whitney test.

Figure 4a–d showed gender‐related differences in cell area and shape for microglia. While females displayed a higher cell body area than males in both WT (Figure 4a) and *En2* ^*−/−*^ (Figure 4c) mice, the contrary was observed for SF (*p* < 0.0001; Figure 4b,d). Analogously, genotype‐related differences are shown in Figure 4‑e–h): microglial cell body area was significantly higher in *En2* ^*−/−*^ mice in both males and females (*p* < 0.0001; Figure 4e,g) while the contrary was observed for SF (*p* < 0.0001; Figure 4f,h).

## 4 DISCUSSION

Given the prevalence of males in ASD and sex‐dependent differences of glial cells in different brain disorders (Retico et al., 2016; Xiong et al., 2023; Yanguas‐Casás et al., 2018), our aim was to analyze changes in *En2* ^*−/−*^ compared with WT mice in both males and females.

Taken together, our findings show that sex‐linked differences can be normally found in mice cerebellum. Specifically, when males were compared with females in the WT group, three out of the four examined cell types showed statistically significant differences.

Sex‐related differences in brain organization have been described in nonpathological mammals; for instance, in rats, the medial preoptic nucleus, the bed nucleus of the stria terminalis, and the medial amygdala displayed a larger volume in males compared to females (Gorski et al., 1978; Hines et al., 1992). In the CA3‐hippocampal region of WT male mice, a higher microglia density was found compared with females (Guneykaya et al., 2023). In humans, neuroimaging studies have indicated differences in grey matter volume between males and females in various cortical areas (Kaczkurkin et al., 2018; Ruigroket et al., 2014), similar results were reported in the mouse brain (Qiu et al., 2018). Furthermore, in the human posterior cerebellum, the volume of gray matter is greater in males than in females (Tiemeier et al., 2010) except for Crus II where the volume is greater in females (Steele & Chakravarty, 2018).

Data from the literature indicate that sex difference in glial number and morphology is dependent upon parameters such as animal species, brain region, and age. In female rats, the number of microglia cells is higher in various brain areas at P30 and P60, whereas at P4, the values are higher in males (Schwarz et al., 2012). In C57BL/6J mice at 13 weeks, the cell density and soma size of Iba1‐ir microglia were highest in the male hippocampus, cortex, and amygdala, whereas at 3 weeks, the highest density was observed in the female amygdala, with no reported differences in the cerebellum (Guneykaya et al., 2018). Previous studies have also reported a sexual dimorphism in GFAP‐ir astrocytes in various brain regions; a denser GFAP immunostaining was observed in the cerebellum and supraoptic nucleus of hamster males, as well as in the posterodorsal portion of the medial amygdala in male rats (Johnson et al., 2008; Suárez et al., 1991, 1992). In contrast, the density of astrocytes in the suprachiasmatic nucleus of the gerbils and in the posterior medial amygdala of rats was higher in females than in males (Collado et al., 1995; Rasia‐Filho et al., 2002). Due to the “physiological” sex differences, it is always convenient to include both sexes in studies aimed at phenotyping mutant mice.

Noteworthy, in *En2*‐mutant mice, the difference in astrocyte and interneuron density observed between WT males and females was not present, while the microglial cell density remained statistically higher in females. Partially in line with our findings, previous molecular and behavioral studies did not report significant differences between male and female *En2* ^*−/−*^ (Brielmaier et al., 2012; Chelini et al., 2019; Sgadò et al., 2013). However, our study reveals that differences may exist in WT animals and thus the lack of differences in mutant subjects might be attributed to gene loss or modification.

Comparing *En2* ^*−/−*^ mice with WT mice, there was a significant increase in astrocyte density in the white matter of the cerebellum in both mutant females and males. Several studies have demonstrated an increase in GFAP protein levels in the brains of autistic subjects, indicating an important role of astrocytes in ASD (for a more comprehensive discussion, see Prata et al., 2017). However, data regarding the number of astrocytes in the human brain are limited and somewhat contradictory. Lee et al. (2017) in the white matter of the prefrontal cortex of autistic subjects reported no change in the density of astrocytes compared with controls, while Vakilzadeh et al. (2022) described a significant decrease in the number of astrocytes in the same brain area. The increase in astrocytes observed in our *En2* ^*−/−*^ model could imply a significant role for these cells in autism, as astrocytes play a crucial role in regulating neurotransmitter homeostasis and, consequently, are essential for maintaining the balance between excitatory (E) and inhibitory (I) neuronal activity (Canitano & Palumbi, 2021). E/I imbalance, specifically a high neuron excitation‐inhibition ratio, appears to be linked to astrocytic activation in the brains of adults with autism (Oya et al., 2023).

The “astrocytosis” observed in both sexes of *En2* ^*−/−*^ mice was accompanied by an increase of microglial cells in females only. Sex‐dependent changes in microglia have been described in the hippocampus of NLGN4^−/−^ mice, a model of ASD, where a decrease in the density of Iba1‐ir microglia has been reported only in males (Guneykaya et al., 2023). In postmortem human subjects (males) with ASD, an increase in microglia was described in various brain areas (Menassa et al., 2017; Tetreault et al., 2012; Vargas et al., 2005), in particular, Vargas et al. (2005) found significantly higher microglial immunoreactivity in the cerebellum of autistic brains.

Microglial cells were also examined for their soma shape: in both sexes, the microglia cell body of *En2* ^*−/−*^ had a larger area and a lower SF (indicating a more jagged shape) compared with the WT phenotype. Controversially, the increase in cell body area may be related to an activation state typical of a pathological condition. On the other hand, a lower SF would suggest a greater roughness compatible with the presence of numerous cellular processes, a morphology commonly found in non‐pathological conditions (Kadlecova et al., 2023).

In the cerebellum of WT females, there were more astrocytes and microglia than in males. Females exhibited a lower microglia cell body SF, indicating a rougher shape associated with a surveillance phenotype, while WT males had a higher SF, suggesting a rounder shape consistent with an activated state (Lenz et al., 2013). These findings, taken together, could support the hypothesis that in WT mice the female cerebellum is more protected than that of males and therefore less susceptible to certain disorders, such as ASD (Liu et al., 2020).

While there is little information related to glial cells in the *En2* ^*−/−*^ mice cerebellum, a recent study reported immune system deficits in the cerebellum of these mice (Pangrazzi et al., 2022). In general, the authors propose a downregulation of pro‐inflammatory pathways even though an increase of the TLR2 mRNA level in *En2* ^*−/−*^ mice was found. This latter finding aligns with the observed increase in astrocytes as the TLR2 receptor IS also expressed by astrocytes (Esen et al., 2004). On the other hand, the increased cell body area of microglia in *En2* ^*−/−*^ mice, as described here, may not be consistent with the reduction of mRNA levels of some pro‐inflammatory molecules reported in *En* mutant (Pangrazzi et al., 2022). However, this data could be in line with the lower cell body SF of microglia, indicating a non‐activated phenotype but rather a state of surveillance (Lenz et al., 2013). The changes in microglia are likely a consequence of the altered homeostasis but not necessarily a specific response linked to a pathological condition (Salter & Stevens, 2017).

The data obtained on interneurons located in the molecular layer do not provide conclusive results: while a significant difference was observed between WT males and females, no differences were observed in the other comparisons. The role of these inhibitory cells is relevant as they modulate the sole output of the cerebellar cortex by shaping the Purkinje firing (Brown et al., 2019). Previous studies have reported a reduction in interneurons in the hippocampus and cerebral cortex of *En2* ^*−/−*^ mice (Sgadò et al., 2013). Furthermore, a decrease in the number of cerebellar Purkinje cells has been observed in both *En2* ^*−/−*^ mice and autistic patients (Kuemerle et al., 1997; Vargas et al., 2005). However, the data reported here did not corroborate these findings as we did not observe significant differences in the density of interneurons (PV‐ir) between *En2* ^*−/−*^ and WT mice.

The precise role of glia in neurodegenerative and neurodevelopmental diseases is still not well understood. However, in recent years, numerous studies have highlighted the connection between glia and neuroinflammation, a common condition in many CNS disorders (Patani et al., 2023; Woodburn et al., 2021). Furthermore, the influence of sex‐related factors is also poorly understood (Lai et al., 2013). Epidemiological data report that ASD is approximately four times more common in males than in females, but most studies were predominantly conducted in male subjects, potentially resulting in a male‐biased understanding of ASD (Lai et al., 2015). The inclusion of equal numbers of males and females in experiments could therefore prove valuable in identifying risk factors or sex‐related protective mechanisms. In this context, our observations could contribute to answering questions about how autism is influenced by sex and glia at the cerebellar level.

## AUTHOR CONTRIBUTIONS

Giulia Lazzarini, Andrea Pirone, and Vincenzo Miragliotta conceived the study; Andrea Pirone, Alessandra Gatta, Francesca Vaglini, Cristina Viaggi, and Giulia Lazzarini performed the laboratory experiments; Andrea Pirone, Vincenzo Miragliotta, and Giulia Lazzarini analyzed the data; Andrea Pirone, Vincenzo Miragliotta, Alessandra Gatta, and Francesca Vaglini drafted the manuscript; Andrea Pirone, Vincenzo Miragliotta, Francesca Vaglini, Giulia Lazzarini, Alessandra Gatta, and Cristina Viaggi revised it critically for important intellectual content. All the authors read and approved the final manuscript.

## CONFLICT OF INTEREST STATEMENT

This research was conducted in the absence of any commercial or financial relationships that could be construed as potential conflict of interest.

## Data Availability

The data that support the findings of this study are available from the corresponding author upon reasonable request.
